# A Case Study Using a Behavioural Contract in Alcohol Dependence within a Crisis Home Treatment Team

**DOI:** 10.1155/2022/6796380

**Published:** 2022-04-19

**Authors:** Andrew John Howe, Cholan Anandarajah

**Affiliations:** South London and Maudsley NHS Foundation Trust, UK

## Abstract

In this case study, we present a novel approach to care within a Home Treatment Team, using a behavioural contract. This is a signed, written agreement that targets specific behaviours for change. The concept draws on social learning theory in that it requires social interaction and a relationship to work. In psychiatric settings, the behavioural contract often finds use in Democratic Therapeutic Communities but rarely in crisis or acute services. In this case study, we attempted to use a behavioural contract within our Home Treatment Team to help a patient address his alcohol dependence and its subsequent effect on his daily living activities. The behavioural contract provided an alternative way to manage a crisis episode. We hope that other crisis service staff reading this case study may use a behavioural contract in their work to a similar beneficial effect.

## 1. Introduction

Crisis Home Treatment Teams were created in the 1980s but did not find their way into National Health Service (NHS) policy until 2000 [1]. They aim to reduce the number and duration of inpatient psychiatric admission and allow services to manage individuals in crisis better. As their name suggests, they do not engage in long term work but attempt to resolve and support patients through a crisis with an expected short duration of treatment. Home Treatment Teams (HTT) are cheaper than inpatient admissions and often have higher patient satisfaction ratings (Ibid.). The HTT in this case study covers a large borough of South London that has a diverse population. It treats between thirty to fifty patients between ages 18-65 at any one time with many different psychiatric diagnoses. Team members are from multiple health care professional backgrounds, including psychiatrists, mental health nurses, occupational therapists, social workers, drug and alcohol specialists, and psychologists. The HTT also signposts to relevant welfare and housing services as these are often critical precipitants to crises.

In this case study, we present a novel approach to care within a HTT, using a behavioural contract. This is a signed, written agreement that targets specific behaviours for change. The concept draws on social learning theory in that it requires social interaction and a relationship to work ([2], Chapter 6). Traditional contracts require that every statement within a contract is objectively verified and that there is an element of reward for success. Behavioural contracts have found use in multiple settings historically, including smoking cessation, hypertension control, lower serum cholesterol, appointment keeping, and adherence with brain and spine injury rehabilitation [3–6]. In psychiatric settings, the behavioural contract finds use in Democratic Therapeutic Communities (DTC) [2]. Here, the community and wish to belong are the underpinnings of the behavioural change in relation to contracts. Within DTC culture, choice-based contracts instead of coercion-based ones encourage responsible agency. Haigh and Pearce suggest that written contracts must contain phrases such as “I undertake to do x/not to do y” (Ibid, p211). If the contract includes a positive injunction, i.e., to do x, the contract must specify the frequency and circumstances. The person to whom the contract applies often proposes it. Within the DTC, all members, including staff and patients, will then consider and vote on the contract. If it is agreed on, then the community will hold the person on the contract to account.

Due to these factors above, behavioural contracts are less likely to be found in acute or crisis services. One can surmise that this is due to the lack of long-term relationships with healthcare professionals over time or the ability to foster a feeling of belonging. The HTT is relatively unique within acute services, be they for physical or mental health, in that treatment is intense, allowing relationships to be built up, and takes place in patients' home environments. Psychologically, one has the feeling of being taken on by a team that can help people in crisis. Given they operate on a consent to treatment basis, it is a team by which patients agree to treatment. This wish to be treated and get out of a crisis period relates to belonging to the team. The loss of the team in this context would therefore be something that patients would wish to avoid. In the following case, we attempted to use a behavioural contract within our HTT to help a patient address his alcohol dependence and its subsequent effect on his daily living activities.

## 2. Case Vignette

The following vignette details are anonymised and changed where not essential to the history to mask identity. The patient discussed has given his consent for the case to be shared in this anonymised format.

Mr. Y is a 58-year-old man who became known to the secondary mental health services following a presentation of low mood and suicidal thoughts in the context of difficulties dealing with his deceased partner's financial affairs with her family members.

Mr. Y was born in the UK with no complications and had no delays reaching his developmental milestones. He grew up with his parents and three siblings with no significant issues during his childhood. Mr. Y struggled academically at school but still achieved a few qualifications. After school, he completed a vocational qualification and worked in his chosen industry for twenty years before changing his profession due to needing to look after his father.

Mr. Y had been in a relationship for 16 years before his partner passed away, six months before his presentation to our team. Following a period of bereavement, Mr. Y initially returned to work. However, he started to have difficulties when they began to deal with his partner's will. As a result, Mr. Y started to become worried about being made to leave the house.

He received a letter from his partner's sister that she was unhappy with him living in the house and using her personal items, which triggered his low mood and subsequent suicidal thoughts. He went on sick leave but started to think about taking an overdose, jumping in front of a train or crashing his car at high speed. Mr. Y contacted his General Practitioner (GP) expressing thoughts of walking in front of a bus and was referred to accident and emergency. Initially, he was discharged back home with advice about counselling services and online cognitive behavioural therapy (CBT).

Mr. Y returned to A&E the following day with ongoing suicidal thoughts and had stopped eating. He reported that he had been consuming four-five cans of beer daily for the previous two weeks. He declined to work with HTT and was placed on the waiting list for informal admission. As a bed was still not available the following day, he was reassessed and he reported sleeping well overnight, and the suicidal thoughts were becoming less intense. As such, the least restrictive option of HTT was rediscussed, and he agreed on this occasion.

The following day, Mr. Y was assessed and accepted for HTT and then medically reviewed the day after and commenced on Mirtazapine. On this occasion, he reported consuming three cans of lager per night, but not on days when he had to work the following day.

He was initially seen daily by various members of HTT, and Mr. Y continued to express fleeting suicidal thoughts. This included seeing members of the psychology team where there were discussions about coping strategies and distraction techniques. He highlighted two goals for the future as getting back to work in the next one-two weeks and tolerating the uncertainty around the probate, which he was aware could take around two years in total.

Mr. Y's presentation worsened as he started to consume more alcohol. He would meet staff intoxicated, show clear signs of self-neglect, and at one point was noted to have injuries to his face from a fall. He was admitted informally to a psychiatric hospital to manage his risk and provide an inpatient alcohol detox. After this short admission, he was discharged back to HTT care. However, he relapsed shortly after discharge and became a dependent user of alcohol again. Given the increase in risk, this caused another admission to a psychiatric hospital to be considered, but we recognised that there was little overall benefit from this intervention previously, and the problem of alcohol use upon discharge would remain. While Mr. Y had contacted local drug and alcohol services, the contact was erratic, and options such as alcohol detox were not available. Transfer to a Community Mental Health Team (CMHT) at that particular time was also unlikely to address his problems. In terms of what a crisis service could offer, we had few options.

## 3. The Behavioural Contract

Given that Mr. Y had been with us for two months already, he was well known to the team members. We felt he wanted to work with us and wanted things to change, but lacked the structure and support. We came to the idea of using a behavioural contract to help Mr. Y reduce his alcohol intake at a rate that was considered safe based on approved guidelines, attend local alcohol services, and attend to his activities of daily living. We drafted a contract that we introduced to Mr. Y, who suggested removing attendance to Alcoholics Anonymous services as he did not wish to use these groups, but agreed to attend other alcohol services. We considered that alternate day contact would be an essential part of the contract as it would help foster a sense of autonomy. The contract (see Box 1) was then instigated with mutual agreement between the team and Mr. Y. We noted that for two weeks, Mr. Y was able to reduce his alcohol intake as per the contract and attend his local alcohol service for further support. He was no longer presenting to the team intoxicated and started to report on the various activities and goals he had achieved between visits. His living environment slowly improved to reflect this change in alcohol use and engagement with services. At the end of the two week contract period, Mr. Y was safe to be discharged back to the care of a CMHT and attend local alcohol services. He reported the contract to be beneficial and stated that he would recommend it to others to motivate change.

Box 1 Behavioural contract.

## 4. Discussion

While the behavioural contract is not a novel creation, we believe its use at a time of crisis and within a crisis service is. Our case illustrates that HTT can develop significant relational attachments to their patients that they can then use to foster change. This contrasts with the common feedback we receive from patient evaluation forms that they see many different staff and do not form therapeutic relationships. While it is true that staff members do change, patients will see the team daily initially, and we inform them that they can call HTT if needed in working hours. Furthermore, we have handovers with the whole team daily where we discuss every patient, so all patients on our caseload are held in mind by the team.

One part of our contract that is different from the usual behavioural contract noted above is that we did not objectively observe Mr. Y's alcohol intake. Practically, this would have been impossible for a team that visits for around thirty minutes at a time. We did not monitor his contact with drug and alcohol services either, choosing to trust Mr. Y's account instead. Besides the practical limitations, we felt this was an appropriate course of action as it made it clear that we were operating from a mutual place of trust. While paternalistic approaches have their place, we supposed that our approach further fostered Mr. Y's autonomy in addressing his drinking. We hoped that this would promote a longer-lasting change in Mr. Y's life. This approach is in line with the use of behavioural contracts in some democratic therapeutic communities. We feel that the presumption of trust between Mr. Y and our team was also significant in the contract's success. There were some concerns from people in other teams that this may be a wasted exercise since we were not paternalistically checking his adherence via other sources. We consider the position of mutual trust can foster autonomy beyond the short time that we used the contract. The results suggest that Mr. Y did not mislead us in his reports about engagement with other services. His presentation also supports his adherence to the contract. When it comes to patient trust, one is reminded of statements around mental capacity that one must assume capacity unless there is evidence to the contrary.

We are working in a time of unprecedented demands on psychiatric services. In our Trust, there is constant pressure on inpatient beds and acute services as a whole. HTT are one solution to the inpatient bed problem, but at times, there is little practical work that can be done, and some patients can become stuck in a crisis episode. If we had not used the contract, then Mr. Y would have been a risky discharge in the context of not being able to meaningfully help him any further or he would have been admitted to a hospital bed. The behavioural contract provided an alternative way to manage a crisis episode. However, we recognise that it would be unreasonable to generalise their usability to every patient. In the case of Mr. Y, we knew him well as a team, and we had many weeks to build up a therapeutic relationship. This is not the case for many patients with HTT, where contact can be more sporadic, or patients do not want to engage with staff deeper than medication provision. Mr. Y's presentation, complicated by alcohol use, is seen reasonably frequently on our caseload. As a team, we will use this intervention again in select cases to see if we can make it a routine part of practice. Given its use in DTCs and their treatment of personality disorder, a behavioural contract may be used in crisis for this population. However, we must balance this with patients becoming attached to a crisis service that cannot hold them in the long term like a community mental health team can. In some cases, the eventual separation can be very distressing and precipitate a further crisis. With these caveats in mind, we hope that other crisis service staff reading this paper may use a behavioural contract in their work to a similar beneficial effect.

## Figures and Tables

**Box 1:**
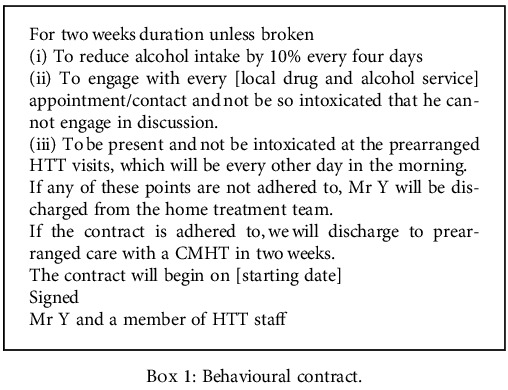
Behavioural contract.

## Data Availability

This paper is a case study and has no underlying data.
